# Editorial: Utilizing big data and deep learning to improve healthcare intelligence and biomedical service delivery

**DOI:** 10.3389/fdata.2024.1502398

**Published:** 2024-10-22

**Authors:** V. E. Sathishkumar

**Affiliations:** Department of Computing and Information Systems, Sunway University, Bandar Sunway, Malaysia

**Keywords:** big data and analytics, deep learning - artificial intelligence, healthcare, intelligent systems and applications, biomedical

The integration of big data and deep learning in healthcare is rapidly transforming the landscape of medical research, diagnostics, and patient care. Our Research Topic, “*Utilizing big data and deep learning to improve healthcare intelligence and biomedical service delivery*,” compiles groundbreaking studies that demonstrate the innovative applications and significant impact of these technologies on healthcare systems worldwide. This editorial synthesizes the contributions of seven featured articles, highlighting their collective advancements in healthcare intelligence.

The primary aim of this Research Topic is to explore and showcase innovative research that harnesses the power of big data and deep learning to address critical challenges in healthcare. By presenting a diverse range of studies, we aim to provide a comprehensive overview of how these technologies are utilized to improve diagnostic accuracy, patient care, and overall healthcare system efficiency.

The exponential growth of healthcare data necessitates advanced analytical tools capable of extracting meaningful insights. Traditional data analysis methods often fall short in managing the volume, variety, and velocity of modern healthcare data. Deep learning algorithms, with their capacity to learn from complex datasets, offer a robust solution to these challenges. These technologies are particularly valuable in personalized medicine, public health monitoring, and clinical decision support, enabling tailored treatments and proactive health management.

Alibudbud's review examines the use of Wikipedia page views as a data source for health research. The study analyzed 29 publications utilizing Wikipedia page views to inform public health services and policies. These studies covered various topics, including non-communicable and infectious diseases, health interventions, and the impact of public events on health information usage. The review highlights the potential of Wikipedia page views to estimate disease incidence, predict public interest in health topics, and improve health education activities. Future research is encouraged to address replication limitations and explore other health topics on Wikipedia.

Li H. et al. introduce DIET-AI, a novel diagnostic system combining dual-channel images and extracted text for diagnosing skin diseases. Developed using a dataset of over 200,000 images and 220,000 medical records from Asian populations, DIET-AI demonstrated diagnostic performance comparable to senior dermatologists across 31 common skin diseases. This study underscores the importance of integrating multimodal data to enhance the accuracy and reliability of AI-driven diagnostic tools, highlighting its potential for clinical application in regions with limited access to specialist care.

Yao and Yang discuss the legal and ethical challenges of using real-world data (RWD) in public health research in China. The article addresses the complexities of balancing personal information protection with the public value of health data. The implementation of the Personal Information Protection Law (PIPL) in 2021 underscores the need for clear guidelines on “separate consent,” cross-border data transfer, and exceptions for scientific research. The authors propose a shift in the legal framework to better support public health research while respecting privacy, essential for advancing RWD use in improving health outcomes and guiding policy decisions.

Li L. et al. present an innovative method for automated segmentation of heart sounds to improve cardiovascular disease prediction and diagnosis. The study designed an audio data analysis tool to segment heart sounds from single heart cycles, validated using a finger oxygen meter. By combining an electronic stethoscope with AI technology, the study achieved accurate identification of heart sounds and murmurs, providing an objective basis for heart sound auscultation and visual display, enhancing the prediction and diagnosis of heart disease.

Chen et al. explore the adoption patterns of mobile apps by doctors for patient communication in Hangzhou and Yancheng, China. The mixed methods study found that social context influences doctors' choice of apps, with doctors in traditional societies favoring social networking apps to maintain social connections, while those in modern societies prefer medical platform apps for reputation marketing. The study provides insights into how societal attributes impact technology adoption in healthcare.

He et al. developed a deep learning algorithm using bilinear convolutional and residual neural networks (BCNN-ResNet) to detect carotid plaques and assess their stability. The study involved training and testing on ultrasound images from multiple hospitals. The algorithm demonstrated high accuracy, sensitivity, and specificity in identifying plaque presence and stability, offering a consistent and objective diagnostic method for carotid artery screening, crucial for stroke prevention.

Alhhazmi et al. employed deep learning (DL) and Bayesian optimization (BOA) methods to predict COVID-19 cases in Saudi Arabia. The study compared the efficacy of BOA and DL, finding that the DL approach, particularly the DQN model, provided more accurate predictions. This research underscores the importance of advanced predictive models in managing public health crises, highlighting AI's role in improving response strategies during pandemics.

The Research Topic “*Utilizing big data and deep learning to improve healthcare intelligence and biomedical service delivery*” showcases significant advancements in healthcare technology. The articles demonstrate the practical applications and potential benefits of integrating big data and deep learning into healthcare systems. By addressing critical challenges and proposing innovative solutions, these studies contribute to the ongoing transformation of healthcare intelligence and biomedical service delivery.

We extend our gratitude to all the authors and reviewers who contributed to this Research Topic. Their dedication and insights have significantly enriched our understanding of how big data and deep learning can revolutionize healthcare.

